# The Association between Malnutrition and Malaria Infection in Children under 5 Years in Burkina Faso: A Longitudinal Study

**DOI:** 10.4269/ajtmh.22-0573

**Published:** 2023-01-09

**Authors:** Elisabeth Gebreegziabher, Clarisse Dah, Boubacar Coulibaly, Ali Sie, Mamadou Bountogo, Mamadou Ouattara, Adama Compaoré, Moustapha Nikiema, Jérôme Tiansi, Nestor Dembélé, Elodie Lebas, Michelle Roh, David V. Glidden, Benjamin F. Arnold, Thomas M. Lietman, Catherine E. Oldenburg

**Affiliations:** ^1^ Francis I. Proctor Foundation, University of California San Francisco, San Francisco, California;; ^2^ Centre de Recherche en Sante de Nouna, Nouna, Burkina Faso;; ^3^ Department of Epidemiology and Biostatistics, University of California San Francisco, San Francisco, California;; ^4^ Department of Ophthalmology, University of California San Francisco, San Francisco, California

## Abstract

The relationship between malaria infection and malnutrition is complex. Using data from a randomized controlled trial of 450 children 0–5 years of age in Burkina Faso, we examined the effect of malaria infection on short-term changes in anthropometric measures, the effect of malnutrition on malaria infection, and whether age modified the effect of baseline anthropometric measures on malaria infection. Malaria infection, assessed by blood smear microscopy and weight, height, mid-upper arm circumference, height-for-age z-score, weight-for-age z-score, and weight-for-height z-score were measured at three time points: baseline, 2 weeks, and 6 months. We used generalized estimating equations adjusted for sex, age, breastfeeding, maternal education, and study treatment (azithromycin versus placebo) for all analyses. Interaction terms were used to assess effect modification by age. Among the 366 children with no malaria infection at baseline, 43 (11.6%) had malaria infection within 6 months. There were no important differences in anthropometric measures at 2 weeks and 6 months between those with and without malaria infection at baseline. There were no significant differences in prevalence of malaria infection by baseline anthropometric measures. Age (0–30 months versus 30–60 months) modified the effect of baseline weight and height on malaria infection. Among those aged 0–30 months, for each kilogram increase in weight, malaria infection increased by 27% (95% CI: 6–53%), and for each centimeter increase in height, it increased by 9% (95% CI: 1–17%), but there were no differences for those aged 30–60 months.

## INTRODUCTION

Malnutrition and malaria continue to be major causes of morbidity and mortality in low- and middle-income countries. Globally, 8.9% of the population in 2019 was undernourished,^1^ and an estimated 241 million cases of malaria were reported around the world.^2^ Africa carries a disproportionate portion of the burden of child malnutrition,^3^ and approximately 95% of all malaria cases are reported from this region.^2^ Children younger than 5 years are especially vulnerable to malaria and malnutrition and account for about 80% of malaria deaths,^4^ whereas nearly half of all deaths in children younger than 5 years are attributable to undernutrition.^4^

The association between malaria infection and malnutrition is complex, and the directionality of these associations is poorly understood.^5^ Studies have shown that malnutrition can increase the risk of infections, illnesses, and mortality in children^4^ through decreased immune function. Conversely, infections can impair food and nutrient absorption by reducing appetite, increasing metabolic requirements, weakening transport of nutrients to tissues, and altering gut lumen, leading to undernutrition.^6^^,^^7^ At a population level, there is evidence that an increase in rates of malaria infection was associated with peaks in admission to therapeutic feeding programs; meanwhile, children with malnutrition also showed a greater risk of complications from malaria, requiring hospitalization.^8^ Although several studies have assessed the relationship between malaria and malnutrition, findings have been mixed. Some studies show that malnutrition had no impact on the risk of malaria infection,^9^^,^^10^ whereas others found that malnutrition increased^11^^–^^13^ or decreased^14^^–^^16^ the risk of malaria. A plausible explanation for the heterogeneity in evidence is that most of these studies were conducted using cross-sectional surveys and thus were unable to establish temporality. Longitudinal studies could provide a more rigorous evaluation of these associations, especially under circumstances where the effects may be bidirectional.^17^ Additionally, increased access to health care coupled with advancements in malaria testing and treatment may affect the duration and severity of malaria episodes^18^ and consequently the effect they have on nutritional status. For instance, one study found that compared with patients admitted during the pre–rapid diagnostic test (RDT) period, patients with malaria admitted to a health center in the post-RDT period had significantly reduced odds of being referred to another health center or receiving antibiotics and a significantly shorter mean length of stay.^18^ A third possible explanation for the heterogeneity of results regarding the association between malnutrition and malaria could be differences in study populations,^9^ with age being a prominent characteristic that could contribute to these differences. Indeed, in areas of high malaria transmission, most clinical malaria cases occur among children younger than 5 years; yet studies have shown that the clinical manifestations and sequalae of infection often differ between infants and toddlers.^19^

To better understand the relationships between malaria infection and malnutrition, we used longitudinal 6-month follow-up data from 450 children 0–5 years of age enrolled in a randomized controlled trial in Burkina Faso. The objectives of this study were to examine the effect of malaria infection on short-term changes in anthropometric measures, the effect of malnutrition on malaria infection prevalence, and whether age modified any of these relationships.

## MATERIALS AND METHODS

### Study design, setting, and population.

For this secondary analysis, we used data from a randomized controlled trial (GAMIN) conducted between 2019 and 2020 to evaluate the potential effect of azithromycin on intestinal microbiome changes and child growth.^20^ The study was conducted in the northwestern region of Burkina Faso in the town of Nouna. Children (*N* = 450) 8 days to 60 months old were recruited. The sample size was determined based on the primary outcome of the trial, which was Shannon’s diversity index of the gut microbiome. A sample size of 450 children (225 per arm) provided at least 80% power for a detectable effect size of 1.02 SDs of Shannon’s diversity with no loss to follow-up.^20^ Children were eligible to participate in the study if their primary residence was in Nouna town, they were able to feed orally, and they were available for the full 6-month study period. Eligible participants were recruited at the Nouna town hospital, which serves all seven sectors of Nouna town. Enrollment began in November 2019, at the end of the rainy season, and the final follow-up visit was in June 2020, prior to the beginning of the rainy season.^20^ Malaria in Nouna town is highly seasonal.^21^ In this population, the prevalence of malaria infection was between 15% and 20% during the first two visits and between 5% and 10% during the 6-month visit.^20^ Thus, all children were enrolled at the end of the high malaria transmission season and were followed up during the low transmission season. The study was reviewed and approved by the institutional review boards at the University of California San Francisco and the Comité National d’Ethique pour la Recherche (National Ethics Committee of Burkina Faso) in Ouagadougou, Burkina Faso. Written informed consent was obtained from the caregiver of each enrolled participant.

### Data collection and measures.

Data were collected at three time points: baseline, 2 weeks, and 6 months after enrollment. Anthropometric measures, including weight, height, and mid-upper arm circumference (MUAC) were taken at each study visit. Weight was measured using the SECA 847 scale (Chino, CA) to weigh infants and children to the nearest 0.1 kg. The scales were calibrated each morning by using a 5-kg weight test. Length/height was measured using a ShorrBoard® (Weigh and Measure, LLC, Olney, MD) to the nearest centimeter. MUAC was measured using a standard MUAC tape. Height-for-age z-score (HAZ), weight-for-age z-score (WAZ), and weight-for-height z-score (WHZ) were calculated according to 2006 WHO standards^22^ to measure stunting (HAZ < −2 SDs), underweight (WAZ < −2 SDs), and wasting (WHZ < −2 SDs). Malaria infection at each time point was determined with blood smear microscopy using a finger prick blood sample. Information on the child’s age, sex, mother’s age, education, and breastfeeding status was collected at baseline from a study staff–administered questionnaire.

### Covariates.

Potential confounders of the association between malnutrition and malaria infection were selected a priori using a directed acyclic graph and based on previous literature.^9^^,^^23^ Covariates that were included in multivariable models were sex, age (in months), breastfeeding status, maternal education, and study treatment (azithromycin versus placebo). Age (in months) was not included in the models that assessed effect modification, as the binary measure of age was included in the interaction term. However, as a sensitivity analysis, we controlled for age in months in the models stratified by age group (0–30 months and 30–60 months) to assess whether a residual effect of age contributed to associations seen within strata of younger and older children.

### Statistical analysis methods.

For all analyses, we used generalized estimating equations (GEEs) with robust standard errors to account for repeated observations in children. Six observations with extreme HAZ, WAZ, or WHZ scores (z-score > 6 or < −6) (*N* = 6) were considered implausible and were thus excluded from analysis.^24^ To understand the effect of malaria infection on changes in anthropometric measures at 2 weeks and 6 months, we compared the mean difference in weight, height, MUAC, HAZ, WAZ, and WHZ between those with and without malaria infection at baseline (expressed as beta coefficients). An interaction term between baseline malaria infection and time was included in the models to evaluate whether baseline malaria infection affected change in anthropometric measures over time. Linear combination (*lincom* command in Stata) was used to obtain the mean difference at 2 weeks and 6 months. Multivariable models were adjusted for sex, age (in months), breastfeeding, maternal education, and study treatment (azithromycin versus placebo).

To examine the effect of baseline anthropometric measures on point prevalence of malaria infection, we restricted our study sample to those who had no malaria infection at baseline (*N* = 366). For this subcohort, we estimated the prevalence ratio of malaria infection at 2 weeks and 6 months for each unit increase in baseline anthropometric measures. GEE models with a binomial family and log link were used to obtain prevalence ratios. The same covariates as above were used in adjusted models for this analysis.

To assess whether the child’s age modified the effect of baseline malnutrition on the prevalence of malaria infection, an interaction term between age (dichotomized as 0–29 and 30–60 months, based on the median age of the study population) and baseline anthropometric measures was included in the GEE models to assess effect modification on the multiplicative scale. The effect of each baseline measure on point prevalence of malaria infection within the strata of age was calculated. We plotted the predicted prevalence of malaria infection with baseline anthropometric measures for each age group to visualize any differences in the malnutrition–malaria infection association in younger and older children.

As a sensitivity analysis, we assessed the study characteristics of those who were lost to follow-up (LTFU) using logistic regression of predictors of missing visits. Predictors included in the model included baseline malaria infection, baseline anthropometric measures, sex, age group, breastfeeding, maternal education, and study treatment (azithromycin versus placebo).

SAS 9.4 (SAS Institute, Cary, NC) was used for data cleaning and descriptive analyses. Stata version 14.2 (StataCorp, College Station, TX) was used for all other analyses.

## RESULTS

Characteristics of study participants are shown in Table 1. Of the final study sample (*N* = 444), 78 participants (17.3%) had malaria infection at baseline. Half (50.9%) of the children were female, and 58.4% were aged 0–30 months, whereas 41.6% were between 30 and 60 months old. Approximately half of the children (51.1%) were randomized to the treatment arm (azithromycin), and 38.1% were breastfeeding. The mean HAZ, WAZ, and WHZ scores at baseline were −0.9, −0.9, and −0.5, respectively (Table 1). Among those with malaria infection at baseline, 61.5% were female, whereas 48.7% of the children without malaria infection were female. Among the 444 children, 21 (4.7%) did not attend the 2 week visit, 57 (12.8%) did not attend the 6 month visit, and 14 (3.2%) missed both the 2 week and 6 month visits. The majority (87.7%) of those who did not attend the 6 month visits did not have malaria infection at baseline, and 64.9% were between 0 and 30 months old.

**Table 1:** Characteristics of children enrolled in the GAMIN trial in Burkina Faso by baseline malaria infection status

Participants (*N* = 444)	All (*N* = 444)	Malaria infection at baseline (*n* = 78, 17.7%)	No malaria infection at baseline (*n* = 370, 82.3%)
Child sex, n (%)			
Female	229 (50.9%)	48 (61.5%)	180 (48.7%)
Male	221 (49.1%)	30 (38.5%)	190 (51.4%)
Mother literate, n (%)	192 (0.4%)	31 (39.7%)	161 (43.6%)
Breastfeeding, n (%)	171 (38.1%)	26 (33.3%)	145 (39.3%)
Treatment, n (%)			
Azithromycin	230 (51.1%)	42 (53.9%)	187 (50.5%)
Placebo	220 (48.9%)	36 (46.2%)	183 (49.5%)
Age (binary)			
0–30 months	263 (58.4%)	48 (61.5%)	215 (58.1%)
30–60 months	187 (41.6%)	30 (38.5%)	155 (41.9%)
Age (in months; mean [SD])	28 (14.5)	29.4 (14.2)	27.6 (14.6)
Height (in centimeters; mean [SD])			
Baseline	85.4 (12.0)	86.4 (11.5)	85.1 (12.1)
2 weeks	86.1 (11.9)	87.1 (11.5)	85.8 (12.0)
6 months	91.4 (11.1)	91.8 (10.4)	91.2 (11.3)
Weight (in kilograms; mean [SD])			
Baseline	11.4 (3.0)	11.5 (3.1)	11.3 (3.0)
2 weeks	11.6 (3.0)	11.9 (3.1)	11.5 (3.0)
6 months	12.6 (2.9)	12.7 (2.9)	12.5 (2.9)
MUAC; mean (SD)			
Baseline	14.3 (1.2)	14.3 (1.4)	14.3 (1.2)
2 weeks	14.3 (1.2)	14.4 (1.3)	14.3 (1.2)
6 months	14.4 (1.1)	14.4 (1.2)	14.4 (1.1)
Height-for-age z-score; mean (SD)			
Baseline	−0.9 (1.3)	−0.9 (1.3)	−0.8 (1.3)
2 weeks	−0.8 (1.4)	−0.9 (1.1)	−0.8 (1.4)
6 months	−0.7 (1.1)	−0.8 (1.1)	−0.7 (1.1)
Weight-for-age z-score; mean (SD)			
Baseline	−0.9 (1.1)	−1.0 (1.1)	−0.8 (1.1)
2 weeks	−0.8 (1.1)	−0.8 (1.1)	−0.8 (1.1)
6 months	−0.9 (1.0)	−0.9 (1.1)	−0.9 (1.0)
Weight-for-height z-score; mean (SD)			
Baseline	−0.5 (1.2)	−0.6 (1.2)	−0.5 (1.2)
2 weeks	−0.4 (1.3)	−0.4 (1.2)	−0.4 (1.4)
6 months	−0.7 (1.0)	−0.7 (1.1)	−0.7 (1.0)

MUAC = mid-upper arm circumference.

### Effect of malaria infection on anthropometric measures.

**Figure 1.**
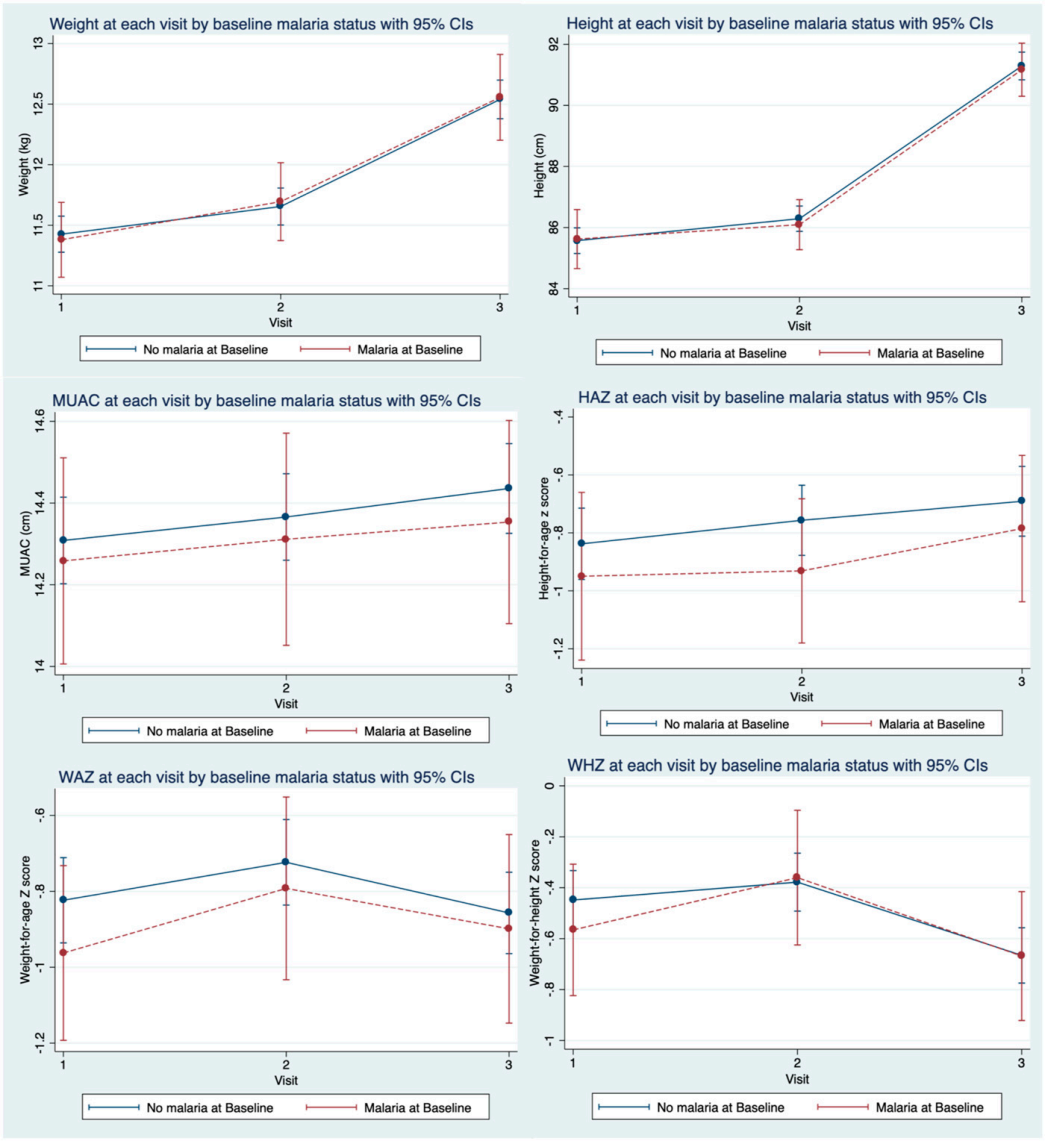
Adjusted anthropometric measures at each visit by baseline malaria infection status. HAZ = height-for-age z-score; MUAC = mid-upper arm circumference; WAZ = weight-for-age z-score; WHZ = weight-for-height z-score.

In the adjusted analysis, children with malaria infection at baseline had slightly lower mean height, MUAC, HAZ, and WAZ at 2 weeks and 6 months after enrollment compared with children without malaria infection, although these estimates did not reach statistical significance (Table 2). As seen in Figure 1, mean weight and height at all time points were highly similar by baseline malaria infection status. However, those with malaria infection at baseline had a lower MUAC and worse HAZ and WAZ at all time points and worse WHZ at baseline but not at 2 weeks and 6 months compared with those without malaria infection at baseline (Figure 1).

**Table 2:** Unadjusted and adjusted mean difference in anthropometric measures at 2 weeks and 6 months by malaria infection status with 95% CI

Malaria (reference group–no malaria infection)	Time point	Unadjusted	Adjusted
Weight (in kilograms)	2 weeks	0.30 (−0.46 to 1.05)	0.04 (−0.32 to 0.40)
	6 months	0.09 (−0.65 to 0.83)	0.02 (−0.37 to 0.41)
Height (in centimeters)	2 weeks	1.04 (−1.80 to 3.89)	−0.19 (−1.12 to 0.73)
	6 months	0.37 (−2.34 to 3.08)	−0.12 (−1.11 to 0.86)
MUAC (in centimeters)	2 weeks	−0.01 (−0.33 to 0.31)	−0.05 (−0.33 to 0.23)
	6 months	−0.07 (−0.38 to 0.23)	−0.08 (−0.35 to 0.19)
Height-for-age z-score	2 weeks	−0.16 (−0.43 to 0.11)	−0.17 (−0.45 to 0.10)
	6 months	−0.08 (−0.36 to 0.2)	−0.09 (−0.37 to 0.19)
Weight-for-age z-score	2 weeks	−0.06 (−0.33 to 0.21)	−0.07 (−0.33 to 0.20)
	6 months	−0.04 (−0.31 to 0.24)	−0.04 (−0.31 to 0.23)
Weight-for-height z-score	2 weeks	0.01 (−0.27 to 0.30)	0.02 (−0.27 to 0.31)
	6 months	−0.01 (−0.28 to 0.27)	0.00 (−0.28 to 0.27)

MUAC = mid-upper arm circumference. Covariates adjusted in models included age, sex, study treatment, maternal education, and breastfeeding.

### Effect of anthropometric measures on prevalence of malaria infection.

Among those with no malaria infection at baseline (*N* = 366), 43 children (11.6%) had malaria infection during follow-up (at 2 week or 6 month visit). In the unadjusted analysis, we found that increases in baseline weight and height were associated with increased prevalence of malaria infection (Table 3). In the adjusted analysis, we did not find a substantial increase or decrease in prevalence of malaria infection associated with an increase in baseline anthropometric measures (Table 3).

**Table 3:** Unadjusted and adjusted prevalence ratios of malaria infection at 2 weeks and 6 months with 95% CI among those with no malaria infection at baseline

Baseline anthropometric measures	Malaria infection at time 2 or 3 (RR, 95% CI)	
	Unadjusted	Adjusted
Weight (in kilograms)	1.11 (1.02–1.19)*	1.05 (0.89–1.24)
Height (in centimeters)	1.03 (1.01–1.05)*	1.02 (0.97–1.08)
MUAC (in centimeters)	1.18 (0.96–1.43)	1.05 (0.81–1.34)
Height-for-age z-score	1.07 (0.87–1.30)	1.05 (0.87–1.26)
Weight-for-age z-score	1.10 (0.91–1.34)	1.10 (0.88–1.37)
Weight-for-height z-score	1.06 (0.88–1.27)	1.07 (0.87–1.31)

MUAC = mid-upper arm circumference. Covariates adjusted in models included age, sex, study treatment, maternal education and breastfeeding.

* *P* value < 0.05.

### Effect modification by age.

**Figure 2.**
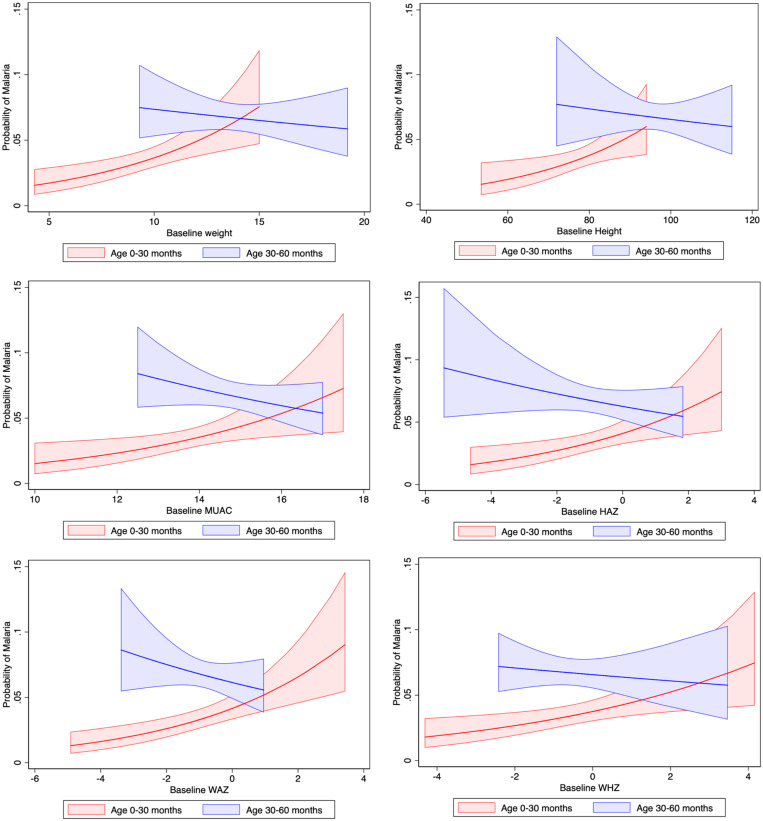
Prevalence of malaria infection by baseline anthropometric measures and age with 95% CIs. HAZ = height-for-age z-score; MUAC = mid-upper arm circumference; WAZ = weight-for-age z-score; WHZ = weight-for-height z-score.

Among those with no malaria infection at baseline, age modified the effect of baseline weight and height on prevalence of malaria infection (*P* < 0.05; Table 4). Among those aged 0–30 months, adjusting for sex, study treatment, maternal education, and breastfeeding, for each kilogram increase in weight the prevalence of malaria infection increased by 27% (RR = 1.27; 95% CI: 1.06–1.53), and for each centimeter increase in height, it increased by 9% (RR = 1.09; 95% CI: 1.01–1.17). For children aged 0–30 months, the prevalence of malaria infection increased with increasing MUAC, HAZ, WAZ, and WHZ, although the CIs for these estimates could not rule out no effect (Table 4). Among those aged 30–60 months, we did not find a substantial increase or decrease in the prevalence of malaria infection associated with an increase in baseline anthropometric measures (Table 4). As shown in Figure 2, as anthropometric measures increased, the prevalence of malaria infection increased substantially for children aged 0–30 months, whereas it decreased slightly for those aged 30–60 months (Figure 2).

**Table 4:** Effect modification of the association between baseline anthropometric measures and malaria infection by age among those with no malaria infection at baseline

Baseline anthropometric measures	Adjusted prevalence ratio and 95% CI by strata of age	
	Age: 0–30 months	Age: 30–60 months
Weight (in kilograms)	1.27 (1.06–1.53)*	0.97 (0.82–1.13)
Height (in centimeters)	1.09 (1.01–1.17)*	0.99 (0.94–1.03)
MUAC (in centimeters)	1.28 (0.92–1.77)	0.90 (0.66–1.22)
Height-for-age z-score	1.24 (0.92–1.66)	0.91 (0.70–1.18)
Weight-for-age z-score	1.27 (0.98–1.64)	0.89 (0.62–1.29)
Weight-for-height z-score	1.18 (0.90–1.55)	0.97 (0.72–1.30)

MUAC = mid-upper arm circumference. Covariates adjusted in models included sex, study treatment, maternal education, and breastfeeding.

* *P* value < 0.05 for interaction term of age and baseline weight and height.

### Sensitivity analyses.

In a sensitivity analysis controlling for age in months in models stratified by age groups (0–30 and 30–60 months), we found that results were very similar with regard to point estimates and statistical significance. The only change was for children aged 0–30 months, where the effect of baseline height on the prevalence of malaria infection changed from borderline statistically significant to borderline insignificant (RR = 1.09; 95% CI: 1.01–1.17 versus RR = 1.08; 95% CI: 1.00–1.17 in sensitivity analysis). In the sensitivity analysis of LTFU, none of the covariates were significantly associated with missing the third or both follow-up visits.

## DISCUSSION

Our findings show that in this study population, baseline malaria infection did not affect growth at measurements 2 weeks and 6 months later. The effect of baseline anthropometric measures on the prevalence of malaria infection varied by child age. An increase in anthropometric measures at baseline increased the prevalence of malaria infection in children aged 0–30 months but not in those 30–60 months. Older children (aged 30–60 months) with lower height, HAZ, and MUAC and younger children (aged 0–30 months) who had higher weight-related measures (i.e., had higher weight, WAZ, and WHZ) had a higher prevalence of malaria infection.

### Effect of malaria infection on anthropometric measures.

Previous studies found mixed results regarding the effect of malaria on growth and malnutrition. Some studies found that malaria could impair growth.^7^^,^^25^ Similar to the studies that did not find evidence for reduced growth among children with malaria,^26^^,^^27^ we did not find a statistically significant difference in anthropometric measures at 2 weeks and 6 months by baseline malaria infection status. However, we found that mean differences in weight, WAZ, and WHZ slightly narrowed at 2 weeks and 6 months between those with and without malaria infection at baseline (Figure 1). This could be due to antimalarial treatment that has been associated with weight gain, which could make up for deficits that may otherwise have been seen among children with malaria infection.^9^^,^^27^ In this study, children who tested positive for malaria using an RDT were given treatment, which could have narrowed the differences in weight-related growth metrics on subsequent visits. Although malaria infection at baseline did not affect growth, those with malaria infection at baseline generally had poorer anthropometric measures, particularly MUAC, HAZ, and WAZ at all time points (Figure 1). This could indicate a poorer nutritional status among children with malaria infection.

### Effect of anthropometric measures on the prevalence of malaria infection.

For the effect of baseline anthropometric measures on the prevalence of malaria infection, in the adjusted analysis, there was no substantial increase or decrease in the prevalence of malaria infection for increasing anthropometric measures. Although the findings from previous studies are mixed, this result is in line with some of the studies that found that anthropometric indices were not important predictors of malaria.^9^^,^^10^ This mix of results could be due to differences in study population, including participant demographics, socioeconomic status, comorbidities, and extent of malnutrition, as well as malaria transmission and season, among other factors that could confound the effect.^25^ The relationship between malnutrition and malaria infection may also have been modified by different factors. For instance, based on our assessment of the effect modification by age, we found that the effect of malnutrition on the prevalence of malaria infection varied by age. Therefore, in the analysis adjusting for age, there may have been no effect of malnutrition on the prevalence of malaria infection, and effects within the different groups may not have been apparent.

### Effect modification by age.

In this study, we found that the effect of baseline anthropometric measures on malaria infection prevalence varied by age. In those aged 30–60 months, there was a slight decrease in the prevalence of malaria infection as baseline measures increased, although the CIs did not reach statistical significance. Contrary to some of the studies mentioned above that found no effect, some studies found that malnutrition increased^11^^–^^13^ or decreased^14^^–^^16^ the risk of malaria. We may have seen a higher prevalence of malaria infection among children with lower anthropometric measures because of weakened immunity, which increases susceptibility to infections. Studies show that inadequate dietary intake could increase the risk for several infections through weight loss, growth faltering, and lowered immunity.^6^ This pattern was also seen in the figure where older children with malnutrition and a particularly lower height, HAZ, and MUAC had a higher prevalence of malaria infection. Older children who were stunted (HAZ < −2), underweight (WAZ < −2), or wasted (WHZ < −2) had the highest prevalence of malaria infection in their age group. Alternatively, although we controlled for maternal education, it is also possible that there could be residual confounding by socioeconomic status and related factors, such as family income, housing, and family size, that may have made children more vulnerable to malnutrition and may have increased the prevalence of malaria infection.^28^

In children aged 0–30 months, lower anthropometric measures were protective of malaria infection (Figure 2). In particular, younger children who had higher weight, WAZ, and WHZ had a higher prevalence of malaria infection. Although there is substantial uncertainty with some of these estimates, these findings reflect important differences in malaria infection between children with larger differences in anthropometric measures. Previous studies suggest that mechanisms by which malnutrition could protect against malaria infection include improved ability of malnourished children to produce certain cytokines in response to stimulation by specific malarial antigens^29^ and potential confounding resulting from improved malaria preventive measures for ill children,^15^ as malnutrition can be accompanied by other illnesses. In particular, because newborns and infants are usually wrapped with clothing and carried on the back of the mother or caregiver in most African countries,^30^ they may have had additional protection from exposure to mosquitoes. One study explored how to use this practice by treating clothing for added protection from malaria.^30^ Therefore, infants in general and those with illnesses and weaker immunity, in particular, may be kept closer physically and cared for differently.

Although the assessment of effect modification was exploratory, differences in the effect of malnutrition on malaria infection by age group may be suggestive of distinct pathophysiologic pathways. Specific micronutrient deficiencies such as vitamin A, zinc, and protein-energy malnutrition have been associated with increased risk of malaria,^31^^,^^32^ whereas some deficiencies (e.g., iron and vitamin E) were found to be protective of clinical malaria in some settings.^32^^,^^33^ Studies show that nutrients can modify aspects of malaria immunity and pathology^32^ and that the association between malnutrition and malaria infection may differ in populations with varying nutritional deficiencies.^25^ Therefore, micronutrient deficiencies may modify the effect of malnutrition on malaria infection, and certain age groups may be more likely to have specific nutritional deficiencies. For example, malnutrition in infants and toddlers who have different feeding habits from those of preschool children may be accompanied by different micronutrient deficiencies, which may lead to varying susceptibility to infections. Particularly, breastfed infants may have different micronutrient deficiencies, as breast milk has lower levels of vitamin D, iron, and vitamin K.^34^ Although we controlled for breastfeeding, which could adjust for differences within the strata of younger children, breastfeeding may partially explain differences across strata, as older children were not being breastfed at the time of the study. Therefore, antibodies, immunity, and specific micronutrients that children acquire from breastfeeding may affect the mechanism or extent by which malnutrition affects their susceptibility to infections, which may be different from that seen in older children with malnutrition. Conversely, age may be considered a proxy for previous malaria infections, which could confer additional immunity^35^ among older kids and potentially affect the mechanism or extent by which malnutrition could affect malaria infection. It may be useful if future studies further explored this mechanism.

### Strengths and limitations.

This study had some limitations. First, we had a relatively small sample size, which could limit our statistical power to detect more subtle differences. However, several other studies, particularly longitudinal studies on this topic, also had similar sample sizes ranging between 202 and 847 participants.^14^^,^^15^^,^^36^^,^^37^ Second, there was loss to follow-up in which 3% of participants did not return after the first visit and 12% did not attend the last visit. A higher proportion of those LTFU were children without malaria infection at baseline. However, based on our sensitivity analysis, none of the covariates were significantly associated with missing the third or both follow-up visits, suggesting that those who remained in the study did not appear to be systematically different from those who were LTFU with regard to study characteristics; however, we cannot rule out the possibility that they may differ by other characteristics that were not measured in the study or by outcome. Additionally, the missing proportions were considerably small and may not pose a threat to validity.^38^ Third, the generalizability of this study may be limited. In this study population, the prevalence of malaria infection was generally low and even lower at the 6 month visit, which was toward the end of the malaria season. Additionally, there was less variability in malnutrition status. On average, children in this study had mild malnutrition. It may be useful if future studies examined these mechanisms in populations with a higher prevalence of malaria infection and in children with moderate and severe malnutrition. Lastly, we cannot rule out that our estimates were subject to residual confounding. Therefore, conditional exchangeability of the groups being compared and identification of a causal effect may not be justified.

This study also had several strengths, including its longitudinal data, which allowed us to establish temporality and comprehensively evaluate bi-directional effects in the same population. This may also be one of the few (if any) studies to assess the role of age in modifying the effect of malnutrition on the prevalence of malaria infection.

## CONCLUSION

The effect of malnutrition on the prevalence of malaria infection may vary by age. The prevalence of malaria infection increased with increasing anthropometric measures among children aged 0–30 months, whereas it slightly decreased for children aged 30–60 months. Children aged 30–60 months with lower height, HAZ, and MUAC and younger children (aged 0–30 months) who had higher weight and WAZ and WHZ z-scores had a higher prevalence of malaria infection. We recommend that future studies examine age-related and other factors that may exacerbate or mitigate the effect of malnutrition on malaria infection. It may also be useful to examine these associations at both individual and community levels.
